# Effects of Different Dentin Surface Cleaning Protocols on Bond Strength of Dual-Cure Resin Cement Following Temporary Cementation

**DOI:** 10.1055/s-0044-1800824

**Published:** 2025-03-12

**Authors:** Vichakorn Vongtavatchai, Boondarick Niyatiwatchanchai, Murali Srinivasan, Thantrira Porntaveetus, Junji Tagami, Anucharte Srijunbarl, Kornwasa Siripamitdul, Dusit Nantanapiboon

**Affiliations:** 1 Department of Operative Dentistry, Faculty of Dentistry, Chulalongkorn University, Bangkok, Thailand; 2 Clinic of General-, Special Care-, and Geriatric Dentistry, Center for Dental Medicine, University of Zurich, Zurich, Switzerland; 3 Center of Excellence in Genomics and Precision Dentistry, Department of Physiology, Clinical Research Center, Faculty of Dentistry, Chulalongkorn University, Bangkok, Thailand; 4 Geriatric and Special Patients Care International Program, Faculty of Dentistry, Chulalongkorn University, Bangkok, Thailand; 5 Department of Cariology and Operative Dentistry, Division of Oral Health Sciences, Graduate School of Medical and Dental Sciences, Institute of Science Tokyo, Tokyo, Japan; 6 Dental Material Research and Development Center, Faculty of Dentistry, Chulalongkorn University, Bangkok, Thailand; 7 Metallurgical Structure Testing and Inspection Center, Iron and Steel Institute of Thailand, Bangkok, Thailand; 8 Center of Excellence for Dental Stem Cell Biology and Center of Excellence and Innovation for Oral Health and Healthy Longevity, Faculty of Dentistry, Chulalongkorn University, Bangkok, Thailand

**Keywords:** air polishing, bond strength, contamination, dentin, MDP-based cleaner, temporary cement

## Abstract

**Objective:**

The aim of this study was to compare the effectiveness of various cleaning protocols on the bond strength of dual-cure resin cement following temporary cementation.

**Materials and Methods:**

Fifty-two human third molars were sectioned to expose superficial dentin and divided into four groups: (1) fresh, noncontaminated dentin (control); (2) pumice cleaning; (3) pumice + sodium bicarbonate air polishing; and (4) pumice + 10-methacryloyloxydecyl dihydrogen phosphate (MDP) based cleaner. Groups 2, 3, and 4 were treated with noneugenol zinc oxide temporary cement, followed by their respective cleaning protocols. After the teeth were cleaned, the restorative procedure was performed. All dentin surfaces were then bonded with a composite restoration using dual-cure resin cement. Shear bond strength was tested using a universal testing machine until failure. Surface morphology was assessed using a scanning electron microscope (SEM), and energy-dispersive X-ray spectroscopy (EDS) was used to analyze the residual elements on the dentin surface.

**Statistical Analysis:**

Group differences were analyzed using one-way analysis of variance (ANOVA), followed by Tamhane's post hoc test. Chi-squared tests were used to assess the differences in failure mode proportions among groups. All statistical analyses were conducted at a significance level of *p* < 0.05.

**Results:**

ANOVA revealed significant differences in bond strength among the groups ( *p* < 0.001). Post hoc analysis showed no significant difference in bond strength between the control group and the sodium bicarbonate air polishing or MDP-based cleaner groups. However, the pumice polishing group exhibited a significantly lower bond strength compared to all other groups ( *p* < 0.001). SEM-EDS analysis confirmed incomplete removal of temporary cement with pumice polishing, as evidenced by residual cement and elevated levels of zinc and oxygen ions.

**Conclusion:**

Pumice polishing alone was insufficient for removing temporary cement, resulting in reduced bond strength of the subsequently applied resin cement. This study demonstrated that combining pumice with sodium bicarbonate air polishing or MDP-based cleaner effectively removed cement and restored bond strength to levels comparable to fresh, noncontaminated dentin.

## Introduction

Bonded restorations have become increasingly prominent in restorative dentistry. This shift in dental restorative practice prioritizes a minimally invasive approach, aiming to preserve the quality of sound tooth structures and restore only the defective areas. 1 This change is driven by the development of adhesive systems with high bond strength that can replicate the bonding area of the dentin–enamel junction 2 and by innovations in glass ceramics and zirconia that have led to high-strength materials requiring minimal tooth preparation while effectively bearing occlusal loads. 3 However, previous publications revealed that the success of bonded restorations was influenced by numerous factors and involves significant technical sensitivities that clinicians must consider. 4 These factors included case selection, 5 choice of materials, 6 tooth preparation procedures, 7 and, importantly, adherence to proper bonding protocols. 4

In the cases using conventional impression techniques, clinicians typically place provisional restorations cemented with temporary materials while waiting for the fabrication of indirect restorations, which usually requires at least a second visit. These provisional restorations are crucial as they alleviate pain and sensitivity while maintaining periodontal health; however, the choice of temporary cements is critical, as some can adversely affect the bond strength of permanent restorations. The results of previous publications showed that eugenol-containing temporary cements interfered with resin cement polymerization, thereby weakening the bond strength. 8 Additionally, residues of temporary cements left on dentin surfaces could interfere the adhesion of permanent restorations, presenting challenges for complete removal and compromising the final bond strength. 9

Currently, researchers are exploring various mechanical and chemical procedures to decontaminate residual temporary cement and enhance bond strength. These methods include tooth prophylaxis with pumice, air polishing with sodium bicarbonate particles, and chemical decontamination. However, some techniques have demonstrated negative impacts on bond strength. For instance, alumina sandblasting, with its higher Mohs hardness compared to dentin and enamel, has raised concerns about excessive abrasion. This excessive abrasiveness can potentially create an interfacial gap between the restoration and the tooth structure. 10 Particles with lower abrasiveness, such as sodium bicarbonate, are proposed due to their lower Mohs hardness, minimizing the risk of dentin surface damage. 11 However, there is no evidence supporting the efficacy of air polishing with sodium bicarbonate in removing noneugenol temporary cement. In contrast, in chemical cleaning procedures, several 10-methacryloyloxydecyl dihydrogen phosphate (MDP) based cleaners consist primarily of MDP-salt and have a mild pH of around 4.5. These cleaners are suitable for both intraoral and extraoral use. Previous studies demonstrated that MDP-based cleaners effectively remove phosphate contaminants from saliva on zirconia-based materials, 12 and eliminate carboxylate cements, 13 thereby improving bond strength.

However, there is limited evidence comparing the effectiveness of different cleaning methods. Therefore, this study aimed to evaluate and compare the most effective protocols for cleaning areas contaminated by temporary cement. The null hypothesis was that there would be no difference in the shear bond strength of dentin when comparing various decontamination methods to the control.

## Materials and Methods

### Teeth Collection and Preparation

Fifty-two intact human third mandibular molars were collected with informed consent prior to the study. The Human Research Ethics Committee of the Faculty of Dentistry, Chulalongkorn University (Study code: HREC-DCU 2023-070) approved the procedures on June 28, 2023. The teeth were disinfected in a 0.5% chloramine-T solution at 4°C for 1 week and were used within 6 months for experiments. Each tooth was examined under a 10X stereomicroscope (SZ 61, Olympus, Japan) to ensure there were no cracks, hypomineralization, or other visible defects.

The teeth were divided into four groups, each containing 13 specimens (10 for the shear bond strength test and 3 for surface morphology and elemental analysis). All selected teeth were embedded in polyvinyl chloride cylinders (SCG, Thailand) with the cement–enamel junction positioned 3 mm above the epoxy resin. A low-speed cutting machine (IsoMet 1000, Buehler) was used to make cross-sectional cuts parallel to the horizontal plane, removing 3 mm from the cusp tips of all specimens to expose superficial dentin. This exposure was confirmed using a stereomicroscope (SZ 61, Olympus, Japan) at 40x magnification. To standardize the smear layer, all surfaces were wet-polished with 600-grit silicon carbide paper (TOA Co. Ltd., Thailand) for 30 seconds using a polishing machine (NANO2000T, PACE Technologies, Arizona, United States) at 100 rpm, according to the protocol established by Santos et al. 14 Following polishing, the specimens were stored in distilled water at 37°C for 24 hours before further processing.

### Temporary Cementation Procedure and Decontamination Protocols

**Fig. 1:**
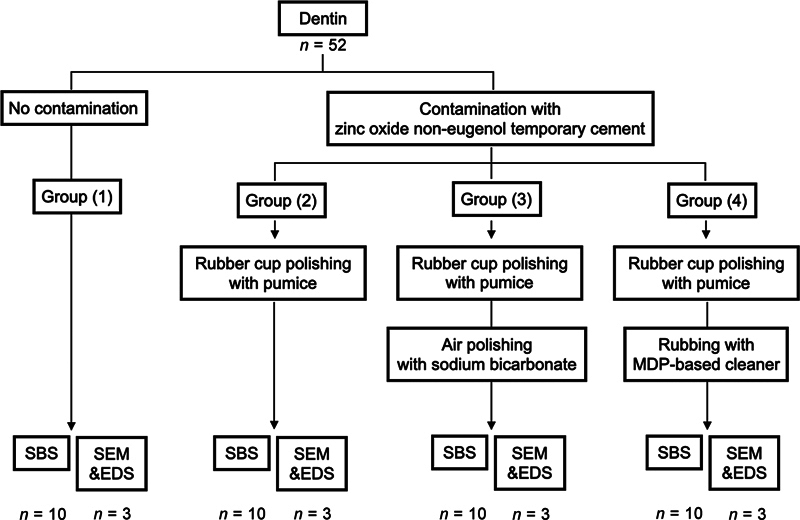
Diagrammatic presentation of experimental groups. EDS, energy-dispersive X-ray spectroscopy; SBS, shear bond strength; SEM, scanning electron microscope.

The details of the noncontaminated group (control group; group 1) and the contaminated groups with different decontamination protocols (experimental groups: groups 2, 3, and 4) are provided in Fig. 1 . All materials used are listed in Table 1 . All preparation steps were performed by a trained dentist to ensure consistency with simulated clinical procedures.

**Table 1:** Materials' detail, composition, and manufacturer's instructions

Material	Product	LOT	Composition	Manufacturer's Instructions
Zinc Oxide Non-Eugenol Temporary Cement	3M RelyX Temp NE	9974658	Zinc oxide, white mineral oil, petrolatum	Mix equal amounts of the base and catalyst pastes for approximately 30 s
Self-cure acrylic	UNIFAST II, GC Corp	2303081	Powder: MMA (methyl methacrylate) and EMA (ethyl methacrylate) copolymer Liquid: MMA	1. Dispense the powder in a rubber cup and add liquid 2. Mix with a plastic spatula for 10–15 s 3. Pour the mixture into the mold, and leave it to cure for 5 min
Air polishing	AquaCare Twin Air Abrasion Restorative Unit (Velopex, London)	10024	Sodium bicarbonate superfine particle size (65 µm)	Angle 45 degrees approximately 4 mm away from the surface at 58 psi for 10 s
MDP-based cleaner	Katana cleaner, Kuraray Noritake Dental	1D0038	MDP (10-methacryloyloxydecyl dihydrogen phosphate), triethanolamine, polyethylene glycol, accelerator, dyes, water	1. Apply the solution to dentin 2. Rub the surface for 10 s 3. Rinse thoroughly, and then dry
Tooth primer	Panavia V5 Tooth primer, Kuraray Noritake Dental	360129	pH 2.0, 10-MDP, original multifunctional monomer, new polymerization accelerator, HEMA (2-hydroxyethyl methacrylate), water, stabilizer	1. Apply and leave primer for 20 s 2. Air-dry
Resin cement	Panavia V5 Paste: automix type, Kuraray Noritake Dental	7R0247	Bis-GMA (bis-phenol A glycidyl methacrylate), TEGDMA (triethylene glycol dimethacrylate), aromatic multifunctional monomer, aliphatic multifunctional monomer, new chemical polymerization accelerator, DL-camphorquinone, photopolymerization accelerator, surface-treated barium glass, fluoroaluminosilicate glass, fine particulate filler	1. Place automixed pastes 2. Light cure for 20 s
Resin composite	Filtek Z350XT 3M/ESPE	10129242	Organic phase: UDMA (urethane dimethacrylate), Bis-GMA, Bis-EMA (ethoxylated bisphenol-A dimethacrylate), TEGDMA Inorganic phase: silica (20-nm nonagglomerated/aggregated), zirconia (4- to 11-nm nonagglomerated/aggregated and agglomerated), clusters, zirconia/silica aggregated particles (20-nm silica particles combined with 4- to 11-nm zirconia)	Apply increment of 2-mm resin composite and light cure for 20 s

To simulate an antagonist temporary crown, cylindrical acrylic stumps were fabricated for each experimental group, with 13 stumps per group, each measuring 10 mm in diameter and 5 mm in height, using Uni-fast III self-cure resin (GC, Tokyo, Japan) in plastic molds. The stumps were initially set for 5 minutes and then soaked in distilled water for 24 hours to complete the curing process.

**Fig. 2:**
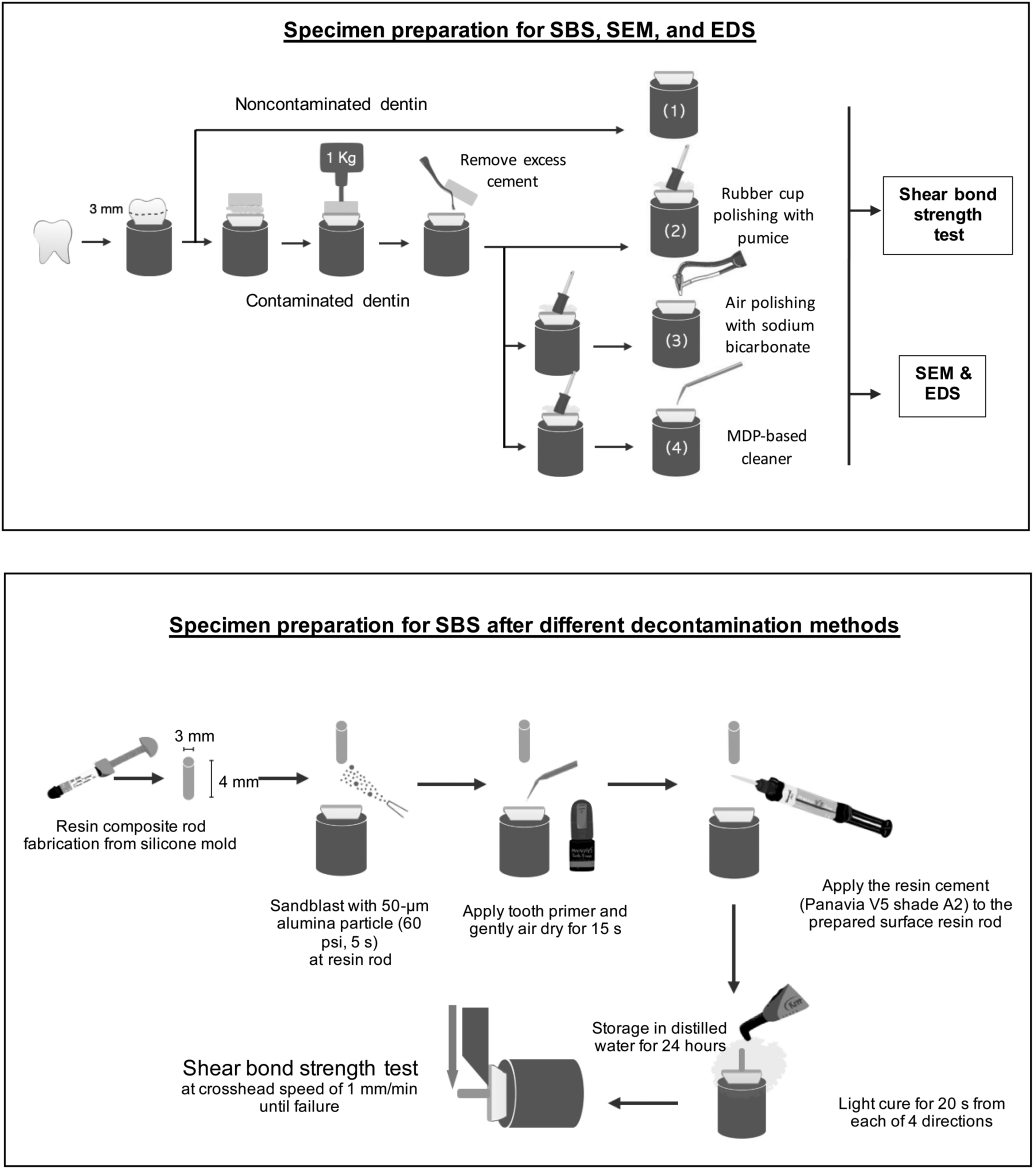
Schematic representation of the procedure for specimen preparation. EDS, energy-dispersive X-ray spectroscopy; SBS, shear bond strength; SEM, scanning electron microscope.

In group 1, specimens were prepared for shear bond strength testing on fresh dentin without any contamination. Specimens from groups 2, 3, and 4 were simulated for temporary cementation with temporary cement (3M RelyX Temp NE Zinc Oxide Non-Eugenol Temporary Cement) on the dentin surface, then compressed with acrylic stumps under a 1-kg load using a test stand (PTC 471 Durometer stand), following the protocol of a previous study. 13 After the temporary cement setting was completed, the specimens with acrylic stumps were stored in distilled water at a controlled 37°C incubator (LK Lab, Korea) for a week, referencing previous studies. 15 Following this period, the acrylic stumps and temporary cement were removed using a spoon excavator with gentle brush strokes, 10 strokes from one side to the other, to ensure complete cleaning as presented in Fig. 2 .

The decontamination protocols for groups 2 through 4 included the following:

- *Group 2:* Specimens were polished with a 5-mg pumice slurry containing 20- to 80-µm irregular-shaped particles (Whip Mix, United States) using a low-speed handpiece (NSK FX Contra 1:1, Japan) set at 1,500 rpm for 15 seconds in a circular motion with a rubber cup, applying a controlled force of 10 N by computer numerical control (IMT, Former A-11, Thailand).
- *Group 3:* Similar to group 2 but followed by decontamination using a chairside air polishing machine (Aqua Care Twin Air Abrasion Restorative Unit, Velopex, London) with 60-µm irregular-shaped sodium bicarbonate particles (ProClean, Velopex, London). The handpiece was positioned 4 mm from the dentin surface at a 45-degree angle and applied at a pressure of 58 psi for 10 seconds.
- *Group 4:* Similar to group 2 but followed by treatment with an MDP-based cleaner (Kuraray Noritake Dental, United States), applying 100 µL to the dentin surface and rubbing with a single-use micro-brush for 10 seconds, per the manufacturer's instructions.

After completing the decontamination protocols, the surfaces were rinsed with an air-water spray from a three-way syringe held 4 cm above the surface for 20 seconds, kept moistened, and immediately subjected to the shear bond strength test.

### Shear Bond Strength Test

Forty resin composite rods, designed to simulate the final restoration, were crafted using Filtek Z350XT (3M ESPE, St Paul, MN, United States) in silicone molds measuring 3 mm in diameter and 4 mm in height. The fabrication process involved layering 2-mm-thick sections of resin composite, each light-cured for 40 seconds using a Demi LED light-curing system (Kerr, Orange, CA, United States) at a controlled intensity of 1,100 to 1,330 mW/cm ^2^ , as measured by a DEMETRON LED Radiometer (Kerr, CA, United States). Additional light curing was performed after mold removal. The rods were then polished using 600-grit silicon carbide abrasive paper (TOA Paint, Thailand) and their dimensions rechecked with a digital caliper (Mitutoyo, Japan) to ensure they met specifications. Prior to the shear bond strength test, the rods underwent sandblasting with 50-µm alumina powder for 5 seconds at a pressure of 60 psi and a distance of 10 mm using a sandblaster (Renfert Rolloblast Blaster, United States).

The shear bond strength tests were uniformly conducted for all groups. Moistened dentin surfaces were treated with 50 µL of Panavia V5 Tooth Primer (Kuraray Noritake Dental) using a single-use micro-brush for 20 seconds, then gently air-dried with oil-free compressed air for 15 seconds. A2 color dual-cure resin cement (Panavia V5; Kuraray Noritake Dental) was then applied to the sandblasted surfaces of resin composite rods via an automix syringe. The rods were pressed onto the dentin surface and compressed with a 1-kg weight to ensure proper seating, and excess resin cement was removed with a single-use micro-brush. Specimens were light-cured for 20 seconds from four directions to ensure complete polymerization and were then stored in distilled water at a controlled 37°C for 24 hours.

The shear bond strength tests were performed using a universal testing machine (EZ-S, Shimadzu, Japan) with a chisel-shaped blade applying shear force parallel to the tooth substrate–adhesive interface at a speed of 1 mm/min until failure occurred. The shear bond strength data, recorded in megapascals, were calculated using the same formulation as described by Santos et al 14 and Arafa et al. 16

To evaluate failure mode, the fracture surface following shear bond strength testing was evaluated under a 30x stereomicroscope (VHX600; Keyence, Itasca, IL, United States). Failure mode classifications were based on three distinct patterns: adhesive, cohesive, and mixed. Adhesive failure was characterized by complete separation of the resin cement from the tooth, leaving no resin cement residue on the dentin surface. Cohesive failure occurs within the resin cement, resin rod, or dentin itself. Mixed failure was identified by partial detachment of the resin cement, where more than 25% of the dentin surface was exposed and remnants of resin cement were visible. 16

### Surface Morphology and Energy-Dispersive X-Ray Spectroscopy Analysis

Following the decontamination protocols, three specimens from both the control and experimental groups were dried and coated with gold, then securely mounted on metal stubs. Surface morphology was evaluated using a scanning electron microscope (SEM; SU3500; Hitachi, Tokyo, Japan) at magnifications of 1,000X, 5,000X, and 10,000X. The pumice and sodium bicarbonate particles were evenly spread over a round glass cover and dried. The sample was then gold coated and examined using an SEM at 200X magnification. Elemental composition, focusing on carbon (C), oxygen (O), phosphorus (P), calcium (Ca), magnesium (Mg), and zinc (Zn), was determined using energy-dispersive X-ray spectroscopy (EDS; EDAX Element EDS, United States) at an acceleration voltage of 10.0 kV.

### Statistical Analysis

Statistical analyses were performed using SPSS software (SPSS, STAT 29 for Mac). The normality of shear bond strength data was evaluated using the Shapiro–Wilk test. Differences among groups were analyzed via one-way analysis of variance (ANOVA) followed by the Tamhane post hoc test. Failure modes were reported as frequencies and percentages, with differences in failure mode proportions among groups assessed using the chi-squared test. A significance level of *p* < 0.05 was applied for all statistical tests.

## Results

### Shear Bond Strength and Failure Mode

The mean and standard deviations of shear bond strength values were presented in Table 2 . The Shapiro–Wilk test confirmed that the data followed a normal distribution, with a significance level of *p* > 0.05. The one-way ANOVA test revealed a statistically significant difference in shear bond strength between the groups ( *p* < 0.001). The Tamhane post hoc test indicated a significant difference in shear bond strength between group 2 and the other groups: groups 1, 3, and 4 ( *p* < 0.001) in all comparisons. Additionally, the statistical analysis showed no significant differences between group 1 and group 3 ( *p* = 0.918), group 1, and group 4 ( *p* = 0.133), and group 3 and group 4 ( *p* = 0.777).

**Table 2:** Shear bond strength values (mPa) of different decontamination methods

Group	Treatment	Mean ± SD
1	Control	12.92 ± 3.12 ^a^
2	Rubber cup with pumice alone	3.87 ± 1.18 ^b^
3	Air polishing with sodium bicarbonate	11.53 ± 3.24 ^a^
4	MDP-based cleaner	10.03 ± 1.83 ^a^

Abbreviations: MDP, 10-methacryloyloxydecyl dihydrogen phosphate; SD, standard deviation.

Note: Values with the same alphabets indicate groups that were not statistically different ( *p* > 0.05).

The most common failure mode observed across all groups was adhesive failure at the cement–dentin interface. The secondary failure mode involved a mixed pattern at the interface, which was evident in all groups. The tertiary failure mode, cohesive failure within the dentin, was only observed in group 3. These findings are detailed in Table 3 . Additionally, the chi-squared test indicated no significant association between the different cleaning methods and failure modes ( *χ* ^2^ = 4.86, *p* > 0.05).

**Table 3:** Failure modes in percentage and *p* -value for decontamination methods

Group	Failure modes (%)			Chi-squared
	Adhesive	Mixed	Cohesive	
1	80	20	0	*χ*^2^ = 4.86 *p* = 0.562
2	80	20	0	
3	50	40	10	
4	70	30	0	

### Surface Morphology and EDS Analysis

**Fig. 3:**
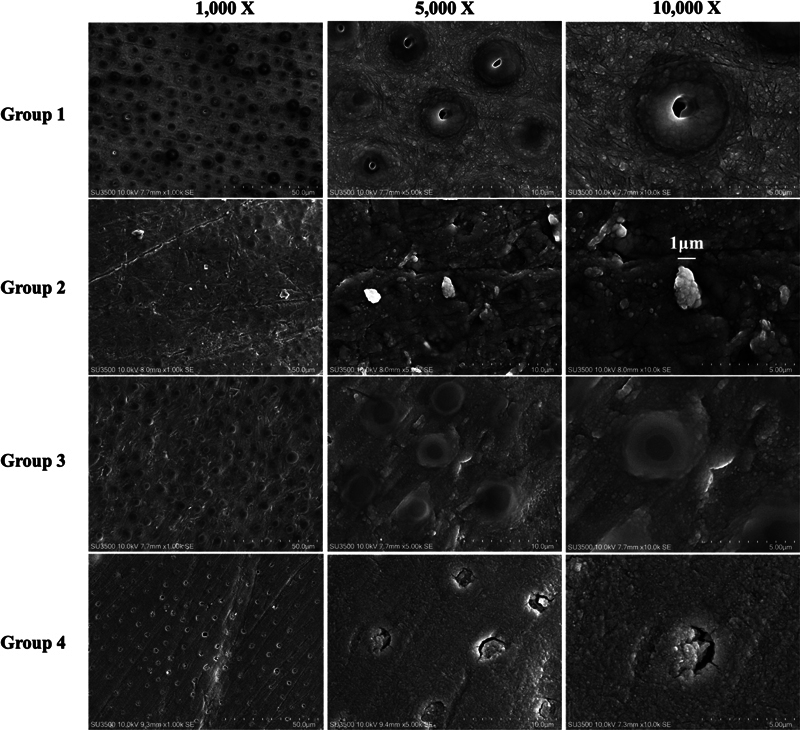
SEM images of dentin surfaces of sound dentin (group 1) and contaminated dentin cleaning after cleaning with different methods (group 2, 3, and 4) at magnifications of 1,000X, 5,000X, and 10,000X.

Surface morphology observations at 1,000X, 5,000X, and 10,000X magnifications revealed distinct differences among the groups, as illustrated in Fig. 3 . In group 1, SEM images demonstrated a smear layer on the surface with no presence of contamination. Group 2 exhibited residues of temporary cement with white irregular globules, approximately 1 μm in diameter, distributed across the surface at all magnifications. In groups 3 and 4, the smear layer partially occluded the dentinal tubules.

**Fig. 4:**
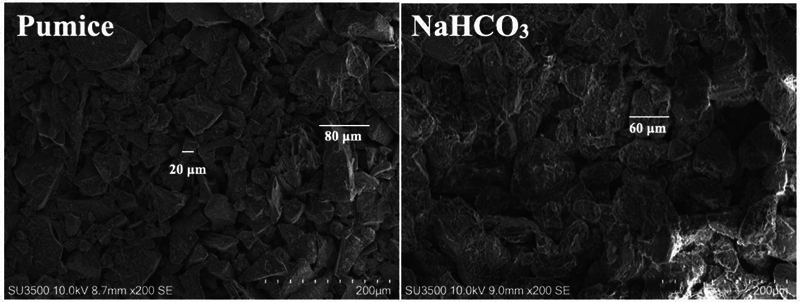
SEM images of pumice and sodium bicarbonate particles.

The SEM images of the pumice and bicarbonate particles, shown in Fig. 4 at 200X magnification, revealed notable differences in morphology. The pumice particles exhibited a combination of small and large irregular shapes resembling spindles, approximately 20 to 80 µm in diameter. In contrast, the bicarbonate particles showed a more homogeneous size distribution with less sharp and more rounded shapes, approximately 60 µm in diameter.

The chemical elements of the main components for each material were presented in Table 4 . All tested materials primarily contained carbon (C), oxygen (O), phosphorus (P), calcium (Ca), magnesium (Mg), and zinc (Zn). The results indicated the highest zinc (Zn) ratio in group 2. In groups 1, 3, and 4, a low level of zinc was still detected on the surface.

**Table 4:** Results of EDS analysis (%weight)

Group	Contamination	Enhancing technique	C	O	P	Ca	Mg	Zn
1	**✗**	None	16.8	25.8	18.90	35.37	1.15	2.47
2	**✓**	None	23.7	13.9	21.03	31.58	0.87	8.84
3	**✓**	Air polishing	11.86	25.72	21.23	36.81	1.36	2.31
4	**✓**	MDP-based cleaner	12.62	25.29	24.63	31.94	1.40	2.48

Abbreviations: C, carbon; Ca, calcium; EDS, energy-dispersive X-ray spectroscopy; MDP, 10-methacryloyloxydecyl dihydrogen phosphate; Mg, magnesium; O, oxygen; P, phosphorus; Zn, Zinc.

## Discussion

The purpose of this study was to evaluate the effectiveness of various cleaning procedures on the shear bond strength between dentin and resin cement. The results showed that polishing dentin with pumice followed by either air polishing with sodium bicarbonate or cleaning with an MDP-based cleaner significantly improved bond strengths compared to using pumice alone. Thus, the hypothesis was rejected.

The presence of temporary cement contamination on dentin has been demonstrated to compromise bond strength of definitive restorations. 17 This is due to a physical barrier of contaminants that impedes the proper infiltration of adhesive resin into the dentin tubules and hindering the formation of a strong and durable bond with the underlying tooth structure. 18 Consequently, restorations placed on contaminated dentin surfaces are more susceptible to compromised bond durability, as reported in *in vitro* studies. 19 Dentin decontamination can be accomplished through various methods to improve bond strength. These methods can be categorized into mechanical methods, such as pumice slurry polishing and air polishing with particles, and chemical methods, including the use of substances like chlorhexidine, sodium hypochlorite, and polyacrylic acid. 20

In this study, to mimic clinical conditions, groups 2 through 4 were first polished with pumice before their respective cleansing procedures. Group 2, polished solely with pumice, showed the lowest shear bond strength, demonstrating that pumice alone was inadequate for effective dentin decontamination. In contrast, group 3, treated with air polishing using sodium bicarbonate at a controlled pressure of approximately 58 psi, as recommended by the manufacturer, achieved bond strength values comparable to the control group, with no significant difference observed. This advantage could be attributed to sodium bicarbonate's lower Mohs hardness compared to pumice particles, which primarily contain silicon dioxide and aluminum oxide, 21 as well as its relatively fine and less sharp particles, with a size of 60 µm, as revealed by the SEM images. This characteristic minimizes the risk of excessive iatrogenic abrasion. 11 This result was consistent with a previous report, which demonstrated that decontamination with sodium bicarbonate particles via air abrasion improved bond strength to between dentin and zirconia. 22 Correspondingly, the SEM image after sodium bicarbonate cleaning revealed complete surface cleaning with no remnants of temporary cement on the dentin surface, generating a homogeneous and uniform finish.

An MDP-based cleaner, the chemical decontamination method used in this study, effectively removed temporary cement and improved bond strength, comparable to the control group. The MDP-based cleaner agent typically has a mild pH and consists of MDP and triethanolamine. The MDP molecules feature a unique structure with both hydrophobic and hydrophilic groups, making it a potential cleaning agent for the intaglio surfaces of various indirect restorations, including those made from lithium disilicate, 23 zirconia, 23 resin ceramic, 13 23 24 and dentin. 24 25 The mechanism of the MDP-based cleaner involves the hydrophobic group of the MDP salt binding to contaminants on the tooth surface when applied. This interaction disrupts the surface tension of the contaminants, facilitating their breakdown. The MDP salt then surrounds the fragmented contaminants, allowing them to be removed by rinsing with water. Correlated with the SEM images, which revealed no residual temporary cement on the surface, an unidentified layer was observed covering the dentinal tubules. This layer potentially resulted from a mild etching effect induced by the MDP component. 26 Moreover, previous research indicated that both air polishing with sodium bicarbonate and the MDP-based cleaner enhanced dentin wettability. 13 22 This improvement in wettability can be another factor contributing to the increased adhesion between resin and dentin. 27

The SEM images demonstrated that surfaces cleaned with sodium bicarbonate air abrasion and the MDP-based cleaner, after tooth polishing, exhibited cleanliness with no residual cement remaining, comparable to the noncontaminated group. This observation corresponded with the high shear bond strength values, indicating the effectiveness of these cleaning methods. However, decontamination with only pumice polishing proved insufficient due to the presence of residual temporary cement. Elemental analysis further confirmed an increase in zinc and oxygen on the dentin surface in the pumice group, indicating incomplete removal of the temporary cement. 13 24 Previous studies revealed that zinc ions had a potential inhibitory effect on the curing of resin materials. 28 Therefore, using more effective cleaning methods, such as sodium bicarbonate or MDP-based cleaner, is crucial to ensure optimal bonding performance.

Air polishing with sodium bicarbonate and the use of an MDP-based cleaner are both effective methods for decontaminating dentin. The MDP-based cleaner is particularly advantageous as it cleans both tooth structures and restorations effectively. Nevertheless, in clinical applications, the air polishing method requires careful management of factors such as particle type, air pressure, duration, and distance to ensure optimal results and avoid potential side effects. 29

In this study, a dual-cure resin cement was classified based on its polymerization mechanism and as a self-etch cement due to its adhesive properties. However, self-etch and self-adhesive resin cements, such as Panavia V5, were more susceptible to contamination compared to total-etch resin cements. 20 The absence of an etching step in these systems led to residual contaminants on the tooth surface, potentially compromising both bond strength and the longevity of the restoration. Conversely, total-etch resin cements, which required phosphoric acid to remove the smear layer, could also remove contaminants, making the negative effects of self-etch systems more pronounced than those of etch-and-rinse resin cements. 18

To further address contamination issues, immediate dentin sealing (IDS) has emerged as a valuable approach by applying a precured dentin adhesive immediately following tooth preparation to generate a hybrid layer that protected against the adverse effects of temporary cement. Meta-analyses have confirmed that the bond strength of resin-coated dentin remains unaffected by temporary cement. 20 However, when resin-coated dentin becomes contaminated and cleaning is required, air polishing with sodium bicarbonate is recommended, as it does not compromise the thickness of the resin layer. 30 Additionally, chairside computer-aided design and computer-aided manufacturing (CAD/CAM) systems, which facilitated same-day restoration delivery, can eliminate the waiting period during restoration fabrication, thereby reducing the risk of contamination from temporary cement.

This study had some limitations, as it focused on the immediate bond strength of restorations to dentin, and future research should consider evaluating bond strength under simulated aging conditions, such as thermocycling or water storage. Additionally, comparing the effectiveness of MDP-based cleaner, air polishing with other particles, and other chemical decontamination methods, such as phosphoric acid etching followed by sodium hypochlorite, would provide valuable insights.

## Conclusion

Pumice polishing by itself proved inadequate for complete removal of temporary cement, leading to reduced bond strength of the resin cement applied afterward. The findings of this study highlight that combining pumice polishing with either sodium bicarbonate air polishing or MDP-based cleaner significantly improved the removal of temporary cement. These methods restored bond strength to levels similar to those achieved with fresh, noncontaminated dentin, suggesting their effectiveness in clinical scenarios where temporary cement must be thoroughly removed prior to final cementation.
